# Knowledge, Attitude, and Practice Regarding Ebola Virus Disease and Related Factors among International Students of Tehran University of Medical Sciences, 2015

**Published:** 2018-05

**Authors:** Kabir Ozigi ABDULLAHI, Kourosh HOLAKOUIE-NAIENI, Abbas RAHIMI FOROUSHANI, Elham AHMADNEZHAD, Owais RAZA, Shahrzad NEMATOLLAHI

**Affiliations:** 1. Dept. of Nursing, Tehran University of Medical Sciences, Tehran, Iran; 2. Dept. of Epidemiology and Biostatistics, School of Public Health, Tehran University of Medical Sciences, Tehran, Iran; 3. Dept. of Health in Disasters and Emergencies, School of Public Health, Tehran University of Medical Sciences, Tehran, Iran

## Dear Editor-in-Chief

Ebola Virus Disease (EVD) is a dreaded, highly virulent infectious disease with a fatality rate 25%–90% (1). It has broken the pattern of all other outbreaks. In view of the unprecedented spread of the EVD and deteriorated consequences on communities, WHO on 8 Aug 2014 declared EVD outbreak as a Public Health Emergency of International Concern (PHEIC). There is no licensed pharmacological agent or vaccine for the treatment and prevention of EVD. There is high need for the control of outbreaks; this requires appropriate preventive measure, accurate and rapid diagnosis of cases (1, 3). The careful practice of hygiene remains the mainstay to prevent transmission of EVD. The emergence and spread of this disease were accompanied by myths, misconceptions, and misinformation about the disease (1). ‘However, WHO has emphasized the need to improve EVD-related health information to dispel misconceptions and myths. The adopted strategies would mitigate fear, prevent stigma and discrimination, and ultimately halt the current and future outbreaks of EVD” (2).

This study was aimed to ascertain the knowledge, attitude, and practice regarding this issue among international students of Tehran University of Medical Sciences (TUMS). Medical and paramedical students as future health workers, are at potential risk of contracting the disease and promote the transmission by occupational exposures. Since they will become the frontline of the response in their various countries, and their occupational health and safety is critical to the control of the outbreak and the maintenance of the health care workforce during the outbreak crisis. This cross-sectional study was conducted among the international students of TUMS. The sample consisted of all international students whose native languages were not Persian. Other students including some Persian-speaking students of Iranian origin yet admitted through International Campus were excluded from the study.

Overall, 135 participants took part in the study as the participants, for the study was voluntary as their consent was sought after. A self-administered questionnaire (which is anonymous to protect the privacy of the participant) was served to subjects who were present in the school and willing to participate in data collection between (Mar–Apr 2015). However, for those subjects who were not in the country, an electronic questionnaire was administered through their E-mail to obtain data on knowledge, attitudes, and practices about EVD. After the instrument has undergone content validity test using 10 experts and using Cronbach’s alpha to determine reliability. The instrument, using coefficient of Variation ratio (CVR) that validity is 79% and that reliability for knowledge and attitude questions were 71% and 76% respectively. Data were analyzed using SPSS ver. 20 (Chicago, IL, USA). In addition, Principal Component Analysis (PCA) was used to extract dimensions of knowledge, attitude, and practice.

Out of 180 questionnaires distributed among the study participant, 135 were filled and returned making a response rate of 75.0%. Overall, 88 (65.2%) were males and 47(34.8%) were females with the mean ages of 28.9 and 22.2 yr, respectively. Of the 135 of the study participant, 83 (61.5%) had at least moderate knowledge of EVD which ranged from general knowledge, animal reservoir, age group at risk, mode of prevention of the disease. Totally, 109(80.7%) of them have a good attitude which ranges from moderate to high approach. Overall, 129(95.6%) of the participants had good emergency approach and hygienic practices which had great impact on prevention of EVD (Table 1).

**Table 1:** Overall level of knowledge, attitude, and Practice among the international Students of TUMS, 2015

***Variable***	***Low (%)***	***Moderate (%)***	***High (%)***	***Total***
Knowledge level	52 (38.5)	61 (45.2)	22 (16.3)	135(100)
Attitude level	26 (19.3)	95 (70.4)	14 (10.4)	135(100)
Practice level	6 (4.4)	94 (69.6)	35 (25.9)	135(100)

Results of knowledge, attitude and practice were as 61.5%, 80.7%, and 95.6%, respectively. We found a reasonably good knowledge (61.5%) level among medical and paramedical students which was similar to different studies conducted in Sudan (98.3%) (4), Ethiopia (66.2%) (5), and Pakistan among medical students (53%) (6). In addition, 80.7% of the study participants had good general attitude toward EHF prevention, this is in conformity with the previous studies (5, 7).

Ethical considerations were observed as the participants were informed that the result of the study is for research purpose only.
